# Validation of the efficacy of air purifiers using molecular techniques

**DOI:** 10.1371/journal.pone.0280243

**Published:** 2023-01-09

**Authors:** Finja Rausch, Franziska Tanneberger, Ahmed Abd El Wahed, Uwe Truyen

**Affiliations:** Faculty of Veterinary Medicine, Institute of Animal Hygiene and Veterinary Public Health, Leipzig University, Leipzig, Germany; Shanghai Public Health Clinical Center, Fudan University, CHINA

## Abstract

The importance of air purifiers has increased in recent years, especially with the “coronavirus disease 2019” pandemic. The efficacy of air purifiers is usually determined under laboratory conditions before widespread application. The standard procedure for testing depends on virus cultivation and titration on cell culture. This, however, requires several days to deliver results. The aim of this study was to establish a rapid molecular assay which can differentiate between intact infectious and distorted non-infectious virus particles. Feline Coronavirus was selected as model for screening. First the samples were pretreated with enzymes (universal nuclease and RNase cocktail enzyme mixture) or viability dye (propidium monoazide) to eliminate any free nucleic acids. The ribonucleic acid (RNA) from intact virus was released *via* magnetic beads-based extraction, then the amount of the RNA was determined using real-time reverse transcription polymerase chain reaction (RT-PCR) or reverse transcription recombinase-aided amplification (RT-RAA). All results were compared to the infectivity assay based on the calculation of the 50% tissue culture infectious dose (TCID_50_). The nuclease has eliminated 100% of the free Feline Coronavirus RNA, while propidium monoazide underperformed (2.3-fold decrease in free RNA). Both RT-RAA and real-time RT-PCR produced similar results to the infectivity assay on cell culture with limit of detection of 10^2^ TCID_50_/mL. Two UV-C air purifiers with prosperities of 100% inactivation of the viruses were used to validate the established procedure. Both real-time RT-PCR and RT-RAA were able to differentiate between intact virus particles and free RNA. To conclude, this study revealed a promising rapid method to validate the efficacy of air purifiers by combining enzymatic pretreatment and molecular assays.

## Introduction

The disinfection of room air by suitable filter systems has become increasingly necessary particularly during the “coronavirus disease 2019” (COVID-19) pandemic [1]. The main method is based on mechanical hindering of the microorganism particles (inertial, diffusion and interception effects) [2–5]. Based on the efficacy, mechanical filters are classified into: pre (>10 μm), medium (1–10 μm), high efficiency (HEPA, >0.3–1 μm) and ultra-low particulate air (ULPA, 0.12–0.25 μm) filters [4–6]. HEPA filters are widely used, for example, in heating, ventilation and air conditioning (HVAC) systems in medical field or in aviation [7].

Another approach is the direct inactivation of microorganisms, e.g. by ultraviolet-C (UV-C), which has the property of degrading the nucleic acids [8]. UV-C light is used as stand-alone units named flow ultraviolet germicidal irradiation (UVGI) lamps or in combination with mechanical filters [2, 3, 9, 10]. Upper-room UVGI is often used for room disinfection particularly in surgical rooms, where sterility is required [8]. In general, the approval of all kind of air purifiers is subject to high standards before commercialization [6, 11–16]. The general rule for mobile air cleaners is that they should reduce the concentration of viruses in the air of the occupied area by 90% (one log_10_ step) within half an hour when used as intended [17].

The efficacy of all air purifiers is first tested under laboratory conditions. Infectious particles are aerosolized through an air purifier test chamber. Air sampling is conducted upstream and downstream of the air purifier on water-soluble gelatin filter attached to air pump. The differences in infectivity before and after the air purification can be determined using e.g. a cell culture system [18, 19]. A reduction of four log levels in 50% tissue culture infectious dose (TCID_50_) represents a virus titer reduction of 99.99% and is therefore considered effective for air purification [20]. However, the virus titer quantification is very time-consuming and requires a well-equipped laboratory with high sterility condition. Molecular methods such as polymerase chain reaction (PCR) or isothermal amplification technologies like recombinase-aided amplification (RAA) are rapid, sensitive, and specific methods for detecting pathogens [21]. Nevertheless, these techniques cannot distinguish between infectious and non-infectious virus particles as nucleic acid (NA) can persist up to three weeks after cell death [22, 23]. However, a technique to get rid of free NA before proceeding with the extraction step can help in differentiating between NA within intact virus particle or free-floating NA. A NA-intercalating dye such as ethidium monoazide bromide (EMA) or propidium monoazide (PMA) can penetrate distorted cells and binds covalently to NA, when photolyzed with bright visible light at a wavelength of 465–475 nm [24–29]. The NA-EMA/PMA-complex cannot be amplified during PCR [30]. Alternative approach is to degrade NA using nucleases. The benzon nuclease (benzonase) was used for the differentiation between intact and heat inactivated viruses [31]. Benzonase is an endonuclease produced in *Escherichia coli (E*. *coli)* strain W_3110_ that cleaves the phosphodiester bond of RNA and DNA. The cleavage results in short NA fragments of three to five base pairs, which can no longer be amplified during PCR [32–34].

The aim of this study is to develop a protocol to speed up the efficacy testing of air purifiers based on a molecular assay. To distinguish between infectious intact virus particles and inactivated distorted virus particles, the sample was first treated with either PMA or universal nuclease to remove free NA; thereafter, the NA extraction procedure will assure the release of NA from intact particles. Feline Coronavirus (FCoV) was applied as a model virus and both RT-PCR and RT-RAA as molecular detection methods. The established protocol was used to test the efficacy of air purifiers in comparison to the infectivity assay based on the calculation of the TCID_50_.

## Materials and methods

### Virus

A field isolate of FCoV (strain Munich) was used for all experiments. FCoV was propagated in Crandell-Rees Feline Kidney (CRFK) cells (catalogue number RIE 769, Friedrich-Loeffler-Institut, Greifswald, Germany) [35]. Cultivation was performed at 37°C and 5% CO_2_ in Dulbecco’s Minimum Essential Medium (DMEM, high-glucose, w/ stable glutamine, w/ sodium pyruvate (BIOWEST, Nuaillé, France)), supplemented with 10% fetal calf serum (FCS, PAA Laboratories, Pasching, Austria), 1% l-glutamine, 1% nonessential amino acids, penicillin (100 IU/mL; Biochrom, Berlin, Germany), and streptomycin sulfate (100 lg/mL; Biochrom). The cultivation resulted in a virus titer of 10^8.25^ TCID_50_/mL.

### Molecular RNA standard

A synthetic molecular RNA standard (nucleotides 28584 to 29096 of the GenBank accession number DQ010921) was used for generating a standard curve during RT-PCR. For this purpose, a DNA strand was synthetized by Thermo Fisher Scientific GENEART (Regensburg, Germany) and transcribed into RNA using HiScribe T7 Quick High Yield RNA Synthesis Kit (New England Biolabs GmbH, Frankfurt, Germany) following the manufacturer’s instructions. After RNA quantification using Qubit RNA BR Assay Kit from Thermo Fisher Scientific (Regensburg, Germany), a ten-fold serial dilution was prepared. Concentrations of 10^5^, 10^4^ and 10^3^ RNA molecules/μL of the standard were used for RT-PCR.

### PMA

The viability dye PMA was purchased from Biotium Inc., CA, USA. It was already dissolved in water at 20 mM concentration and was stored at -20°C, defrosting was performed directly before use at room temperature. The reaction mixture of 100.5 μL consisting of 0.5 μL of PMA (100 mM) and 100 μL of FCoV RNA was incubated in a dark chamber for 10 min. For photoactivation, the sample was exposed to a blue LED lamp (H-B3, UltraFire, NJ, USA) with a wavelength of 470–475 nm at a distance of 15 cm at 600 rpm for 30 min.

### Nuclease and RNase

The Pierce Universal Nuclease for Cell Lysis (Thermo Fisher Scientific Inc., MA, USA) and the RNase cocktail enzyme mix (Thermo Fisher Scientific Inc., MA, USA), both stored at -20°C, were used in this study. First, the total reaction volume of 56 μL contained 40 μL of rehydration buffer (Jiangsu Qitian Gene Biotechnology Co., Ningbo, China), 5 μL of magnesium acetate (Jiangsu Qitian Gene Biotechnology Co., Ningbo, China), 6 μL of universal nuclease, and 5 μL of sample. In the first step, FCoV RNA equivalent to 10^8^ TCID_50_/mL was tested as representative of free RNA. In a second step, ten-fold serial dilution of FCoV cell culture supernatant (titer of 10^6^ to 10^4^ TCID_50_/mL) was screened as representative of intact virus particle. In a third step, ten-fold serial dilution of FCoV cell culture supernatant (titer of 10^8^ to 10^2^ TCID_50_/mL) enriched with 5 μL of FCoV RNA was tested. The mixture was incubated at 600 rpm and 37°C for 30 min. The enzyme reaction was stopped by adding 5 μL of ethylenediaminetetraacetic acid (EDTA, Sigma-Aldrich Chemie GmbH, Taufkirchen, Germany).

The samples taken during testing of an air purifier were subjected to modified pretreatment with the universal nuclease, the RNase cocktail enzyme mix and a reaction buffer consisting of Tris (50 mM, Carl Roth GmbH + Co. KG, Karslruhe, Germany), magnesium chloride (1 mM, Rapidozym Gesellschaft für Laborhandel und DNA Diagnostika mbH, Berlin, Germany) and bovine serum albumin (BSA, 0.1 mg/ml, Carl Roth GmbH + Co. KG, Karlsruhe, Germany). The total reaction volume of 57.5 μL was composed of 45 μL of the reaction buffer, 6 μL of the universal nuclease, 1.5 μL of the RNase cocktail enzyme mix, and 5 μL of the sample. This was followed by an incubation period at 450 rpm and 37°C for 60 min. The enzyme reaction was stopped by adding 5 μL of EDTA and incubating the mixture at 60°C for 10 min.

### Extraction

RNA extraction was carried out using the Dynabeads SILANE Viral NA Kit (Thermo Fisher Scientific Inc., MA, USA) according to manufacturer’s instructions with few modifications as following. Briefly, the sample volume was 56 μL (reaction mixture containing only the universal nuclease) and 57.5 μL (reaction mixture containing the enzyme mix), respectively, the wash steps were performed for one time, and the RNA was eluted in a final volume of 60 μL.

### Real-time RT-RAA

A previously published protocol for the detection of a highly conserved region of the FCoV 7b gene was used for real-time RT-RAA [36]. RT-RAA Nucleic Acid Amplification Kit (Fluorescent Method, Jiangsu Qitian Gene Technology Co., Ningbo, China) was used. The final reaction volume of 50 μL contained 25 μL of rehydration buffer, 2.5 μL of magnesium acetate, 2.1 μL of forward primer (10 μM/μL), 2.1 μL of reverse primer (20 μM/μL), 0.6 μL of probe (10 μM/μL), 12.7 μL of PCR clean water and 5 μL of extracted RNA. The sequences and designations of the primers and the probe were as follows: FCoV RPA FP1 (forward primer) 5’-TCATCGCGCTGCCTACTCTTGTACAGAATGGTAAG-3’; FCoV RPA RP3 (reverse primer) 5’-ACTAGATCCAGACGTTAGCTCTTCCATTGTTGGCTC-3’; FCoV RPA Probe 5’- ATCTAAACTTCCTAA (BHQ1—dT, Tetrahydrofuran and FAM—dT)GCAATAGGGTTGCTTGTACCTCCTATTACACG-Phosphate. The reaction mixture was added into the lid of the tube which contained freeze-dried reaction-pellets. After closing the tube, it was centrifuged, mixed and centrifuged again. The tube was placed into the T8-ISO device (Axxin, Fairfield, Australia) and incubated at 39°C for 15 min. A mixing and centrifuging step was conducted after 320 s.

### Real-time RT-PCR

A previously published protocol for the detection of a highly conserved region of the FCoV 7b gene was used for real-time reverse transcriptase PCR [37]. QuantiTect Probe RT-PCR Kit (Qiagen, Hilden, Germany) and the Stratagene Mx3005p QPCR system (Agilent Technologies, Santa Clara, CA, United States) were used. The reaction mixture of 20 μL was composed of 10 μL of QuantiTect Probe RT-PCR Master-Mix, 7.5 μL of PCR clean water, 0.2 μL of QuantiTect RT Mix, 0.5 μL of forward primer (10 μM/μL), 0.5 μL of reverse primer (10 μM/μL), 0.3 μL of probe (10 μM/μL) and 1 μL of extracted RNA. The primers and the probe were as follows: FCoV1128f (forward primer) 5`-GATTTGATTTGGCAATGCTAGATTT-3; 5`-FCoV1229r (reverse primer) 5`-AACAATCACTAGATCCAGACGTTAGCT-3; probe 5`-6FAM-TCCgCTATgACgAgCCAACAATggATMR-3`(40). The thermal cycling protocol consisted of a 30 min reverse transcriptase step at 50°C, an initial heat activation step at 95°C for 15 min and 40 cycles at 94°C for 15 s and at 60°C for 45 s.

### Statistical analysis

The t test was used to compare the viability pretreatments with the controls. The data were considered significant with values of p < 0.5. The data analysis was performed with GraphPad Prism (GraphPad Software, CA, US).

### Design of the filter experiment

**Fig 1:**
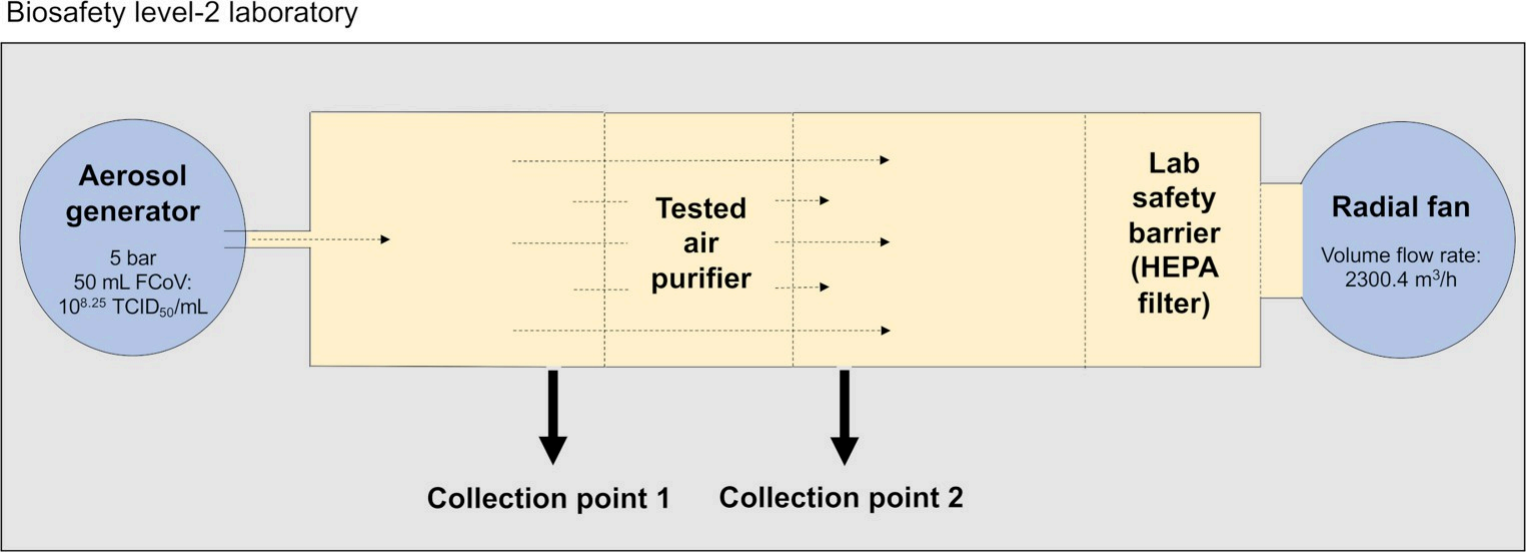
Air purifier testing cabinet.

The test facility setup for the air purifier test was used as described by Wenke et al. (Fig 1) [18]. Two UV-C lamps (2036-4K, 36 Q; sterilAir, Weinfelden, Switzerland and UV-Air Protector, Weidenberg, Hans Prechtl GmbH & Co.KG) were tested. FCoV was aerosolized by an ATM 230 aerosolizer (Topas GmbH, Dresden, Germany) and passed through the air purifier test chamber with a volume flow rate of 2300.4 m^3^/h. Air collection was performed by placing both an air sampler pump (Analyt-MTC GmbH, Müllheim, Germany) and water-soluble gelatin filters (Sartorius 12602-80-ALK, Sartorius AG, Göttingen, Germany) in front of and behind the air purifier. The gelatin filters were dissolved in 5 mL of DMEM at 37°C in 10 min. The modified pretreatment with universal nuclease and RNase cocktail enzyme mix was performed as described above. After RNA extraction, amplification was performed using RT-RAA as well as RT-PCR. Extracted samples without pretreatment served as control. Simultaneously, the samples were analyzed for infectivity by performing virus titration. For this purpose, 100 μL of CRFK cells were added to each well of a 96-well plate. Samples were serially diluted and 100 μL was added to each well of the 96-well plate. The virus-specific cytopathogenic effect (CPE) was assessed microscopically over a period of 6 days. Afterwards, the TCID_50_ was calculated using the Spearman-Kaerber method. The retention efficacy of the air purifier was calculated using the following equation:

$$Reduction\;(\%)=\frac{pathogen\;number\;behind\;the\;air\;purifier}{pathogen\;number\;in\;front\;of\;the\;air\;purifier}*100\%$$

## Results

### Result output of combining nucleases and the molecular assay

**Fig 2:**
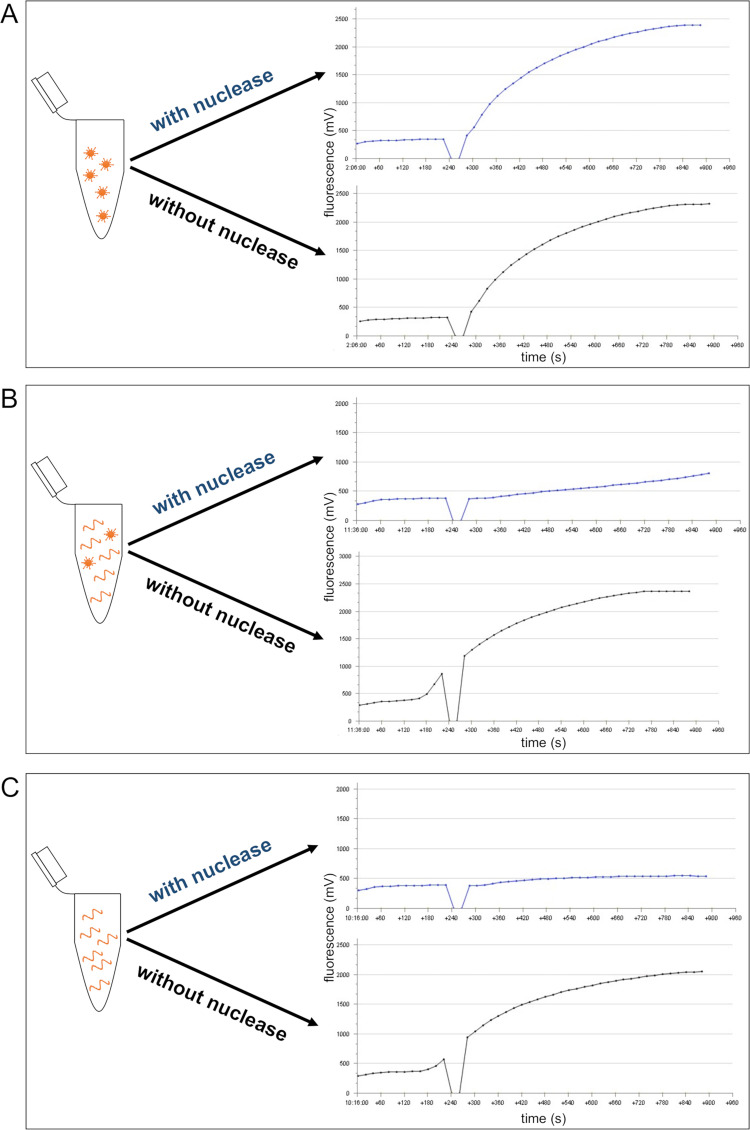
Result outputs by combining nucleases and RT-RAA assay. Pretreatment with nuclease of samples containing only intact virus particles did not interfere with the RT-RAA signals (A). Samples containing both intact virus particles and free NA give different results because the free NA are degraded by the nuclease (B). Samples containing only free NA give no signal when pretreated with nuclease (C).

After adding a nuclease to a particular sample, three result outputs can be delivered. First, samples containing only intact virus particles give the same result, regardless of whether pretreatment with the nuclease was carried out or not (Fig 2A). The second outcome is obtained by samples containing a mixture of intact viruses and free NA. The nuclease degrades the free NA, but the intact viruses can still be detected. Therefore, the RT-RAA signal of the pretreated samples represent the concentration of the intact virus (Fig 2B). As a third option, the nuclease is applied to a sample containing only free NA. No NA was detected by RT-RAA in this case (Fig 2C).

### PMA *versus* universal nuclease

**Fig 3:**
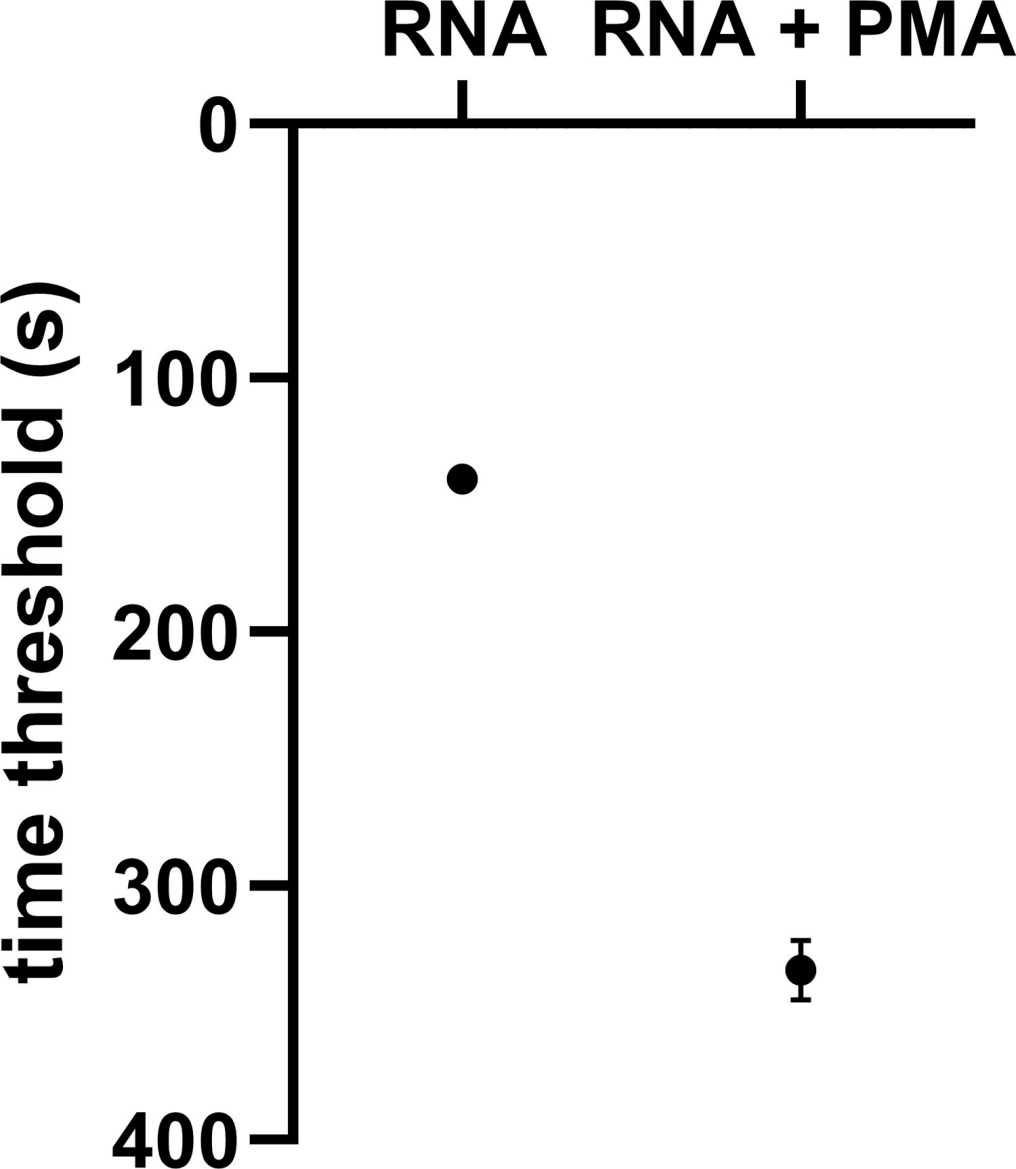
Effect of PMA on free FCoV RNA equivalent to 10^8^ TCID_50_/mL. Delay in time threshold after application of PMA was recorded. The difference between RNA and PMA pretreatment was statistically significant (p < 0.001).

The efficacy of the universal nuclease as well as the viability dye PMA was first tested with FCoV RNA in triplicate. The universal nuclease was able to degrade the entire RNA (Fig 2C). In contrast, only a 2.3-fold delay in the time threshold (tt) was observed on average when using PMA indicating less effectiveness (Fig 3). Due to the low performance of PMA, all further tests were carried out with the universal nuclease.

### Effect of universal nuclease on infectious intact viruses

**Fig 4:**
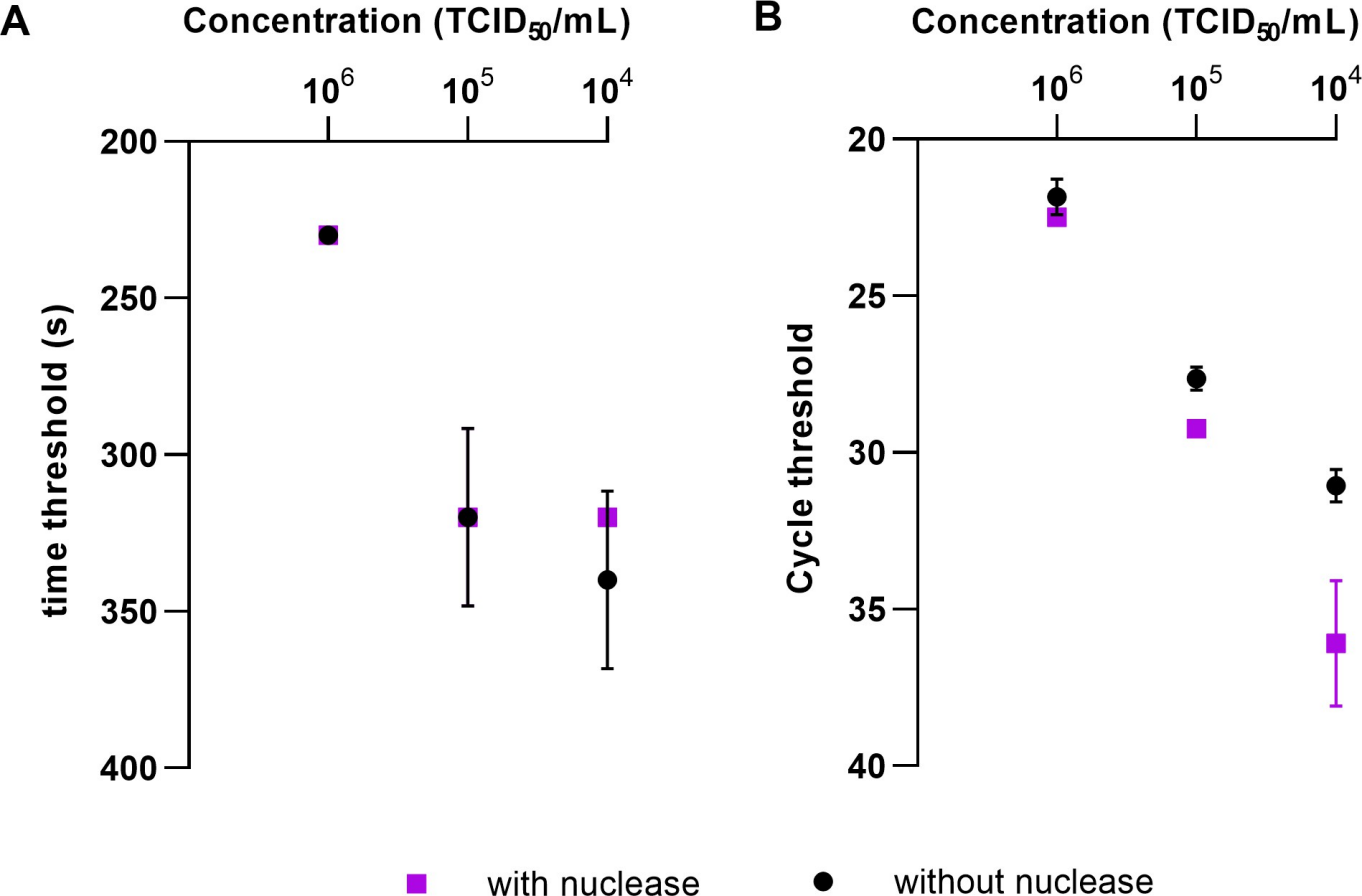
Effect of the universal nuclease on various concentration of infectious intact FCoV viruses. Similar time threshold with or without nuclease was detected (A), however, RT-PCR assay showed a decreased cycle threshold in nuclease pretreated samples, especially samples with lower TCID_50_ (B). No statistically difference was observed between intact virus particles and intact virus particles pretreated with universal nuclease (p = 0.7054 (RT-RAA) and p = 0.6163 (RT-PCR)).

The effect of the universal nuclease on intact virus particles represented by a dilution series of a cell culture supernatant was verified in duplicate. In both RT-RAA and real-time RT-PCR, the dilution series with pretreatment shows almost the same results as the dilution series without pretreatment (Fig 4), which means that the cell culture supernatant contains infectious intact virus particles that cannot be degraded by the universal nuclease.

### Effect of universal nuclease on spiked samples with a combination of intact virus and free RNA

**Fig 5:**
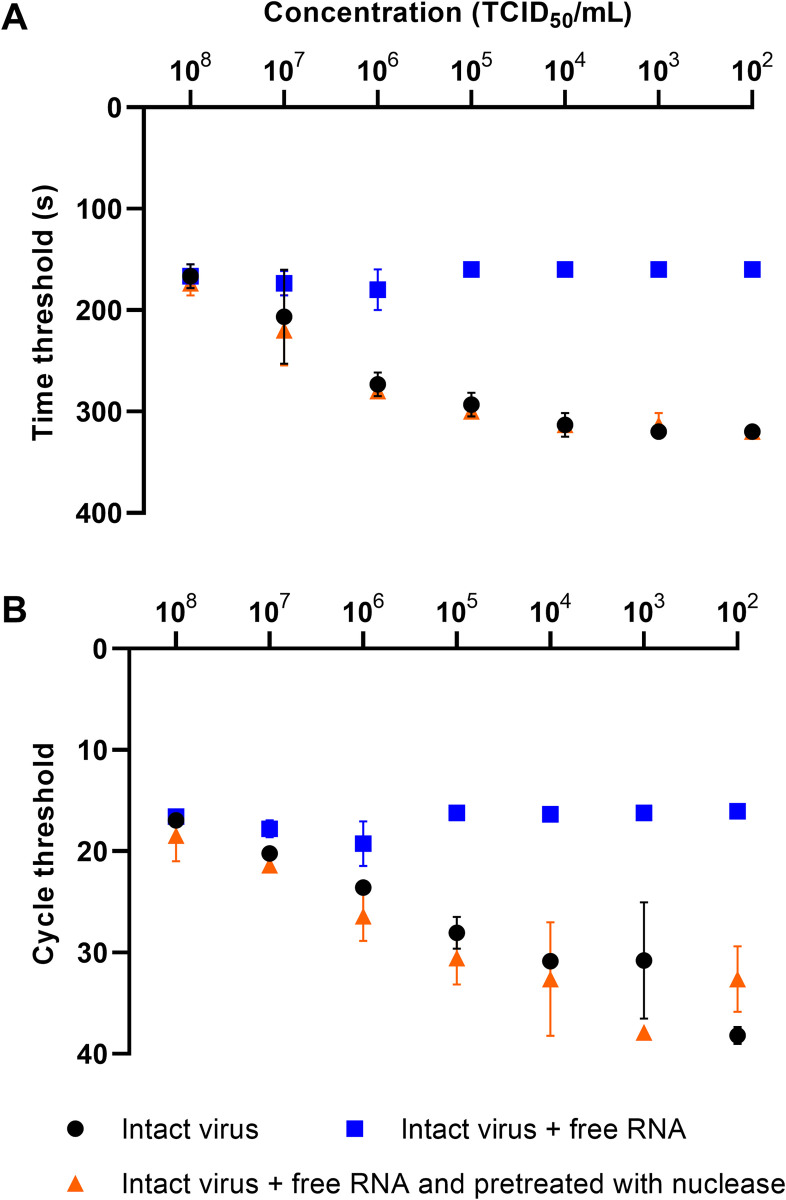
Effectiveness of the universal nuclease on spiked samples with free RNA. In both RT-RAA (A) and real-time RT-PCR (B), the free RNA was degraded when pretreatment with the universal nuclease was performed (orange triangles). The concentrations of those samples correspond to the concentrations of intact virus particles without free RNA and pretreatment; therefore, the orange triangles and black dots overlap. For both RT-RAA and RT-PCR, the differences between intact viruses plus free RNA and intact viruses plus free RNA pretreated with universal nuclease were statistically significant (p < 0.001). The differences between the intact virus particles without enrichment and with enrichment of free RNA were also statistically significant (p < 0.001 (RT-RAA) and p = 0.0035 (RT-PCR)). No statistically difference was recorded between intact virus particles and intact virus particles plus free RNA pretreated with universal nuclease (p = 0.8945 (RT-RAA) and p = 0.6827 (RT-PCR)).

The dilution series of cell culture supernatant (i.e., intact virus particles) was enriched with FCoV RNA. The mixture was pretreated with universal nuclease. In both RT-RAA and real-time RT-PCR, only the RNA was amplified from the intact virus particle as the free RNA was degraded when pretreated with the universal nuclease (orange triangles in Fig 5). In case of samples containing intact virus particles, the same results were obtained indicating the success of the universal nuclease pretreatment (orange triangles and black dots in Fig 5).

### Air purifier test

Two UV-C lamps were tested in the air purifier testing cabinet. The collected samples were pretreated with a mixture of universal nuclease and RNase cocktail enzyme mix. The pretreatments were carried out in duplicate.

**Fig 6:**
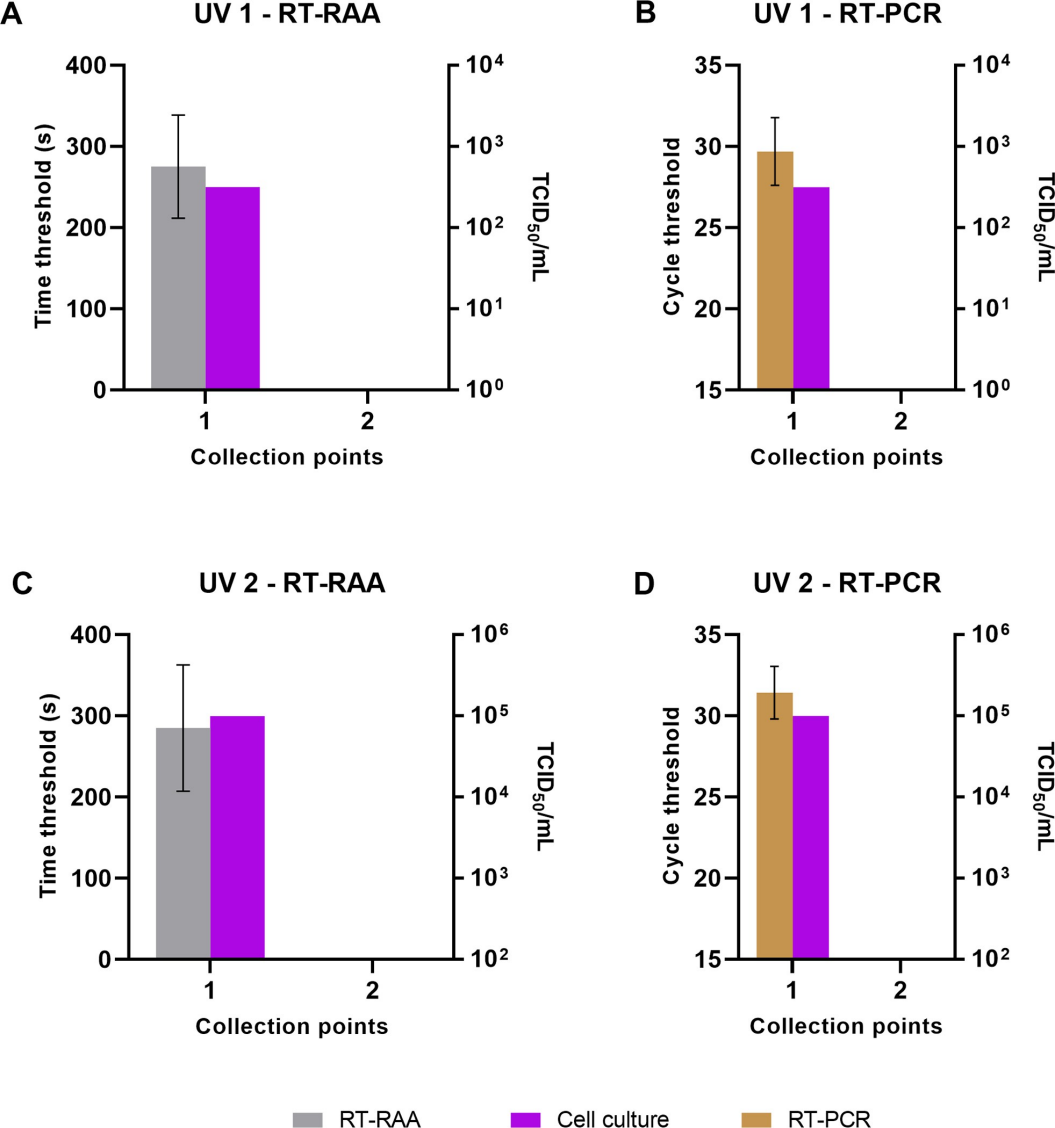
Effectiveness of two UV-C lamps in inactivating infectious virus particles. Collection point #1 represents samples taken in front of the air purifier, while collection point #2 represents samples collected after air purification. Samples collected before the air purification have the expected concentration of viruses in cell culture as well as in RT-RAA and real-time RT-PCR. Enzyme pretreatment of the samples from collection point #2 results in absence of signals in both RT-RAA (A and C) and real-time RT-PCR (B and D).

The first UV-C lamp was able to inactivate the viruses with 100% efficacy. The samples collected in front of the air purifier (collection point #1) resulted in a signal in both RT-RAA (Fig 6A) as well as RT-PCR (Fig 6B), while no signal was detected in the samples collected after air purification (collection point #2). The same results were confirmed on the cell culture. The second UV-C achieved the same results as the first experiment (Fig 6C and 6D). The only difference measured between the two UV-C lamps was the total concentration of the FCoV at the collection point #1 (10^5.75^ TCID_50_/mL and 10^2.5^ TCID_50_/mL, respectively). This indicates that the enzyme mixture works with various amounts of virus particles.

## Discussion

Air purifiers play an important role in prevention of airborne viral diseases and must be tested according to official standards before commercial use. The gold standard method is determining the infectivity using cell culture-based approach, which is time-consuming [18, 19]. In this study, the efficacy of air purifiers was tested using a rapid protocol based on a molecular assay combined with nuclease pretreatment. By combining a RNase cocktail, a universal nuclease and RT-RAA, successful degradation of free RNA was achieved, allowing differentiation between intact infectious and distorted FCoV particles. These results were confirmed by determination of the TCID_50_ in cell culture.

There are several methods to evaluate air purifiers, including animal experiment [38] as well as laboratory-based test chambers [39, 40]. To determine the efficacy of the air purifier, the samples collected upstream and downstream of the air purifier can be tested for the presence of infectious pathogens based on cell culture system. Culturing viruses requires trained personnel, well-equipped laboratory and require at least three days to evaluate the results [41]. In contrast, molecular methods require few hours in case of real-time RT-PCR or 15 minutes for RT-RAA. The real-time RT-PCR must be performed using an expensive thermal cycler, while the RT-RAA was conducted in a mobile suitcase lab [21, 36].

In the present study, a comparison was first made between the viability dye PMA and the universal nuclease. The PMA revealed a poor performance in our set up. PMA proved to be a promising approach for distinguishing between viable and non-viable bacteria [26] and has replaced the viability dye EMA, as the latter also leads to loss of DNA of viable cells and is, therefore, less specific concerning live-dead discrimination [26, 42, 43]. A major drawback of PMA is its inconsistency when applied to viruses [43, 44]. In our study, the PMA was incubated with free RNA, which resulted in a partial elimination of free NA. Similar results have been obtained by others [45, 46]. The poor performance of the PMA with RNA viruses was expected as PMA binds preferentially to double-stranded DNA [47]. In addition, there are many variables that need to be considered upon using PMA, e.g. the dye concentration, the pH and salt concentration of the sample, the type of the light source or the dye incubation conditions [43, 46, 48]. For example, light sources used in the previous studies were inconsistence, therefore, no optimal unified light exposure time is mentioned in the literature [49]. It is not yet fully understood how EMA or PMA prevents the amplification of nucleic acids. One theory is that the DNA is fragmented during irradiation with visible light [50]. Based on this, it is assumed that the length of the amplified target gene plays an important role [49]. In real-time fluorescence-based assay, like in our study, the amplicon size is small, therefore, a limited effect of PMA was observed. The situation is different upon applying conventional PCR with longer amplicon size.

The universal nuclease resulted in complete degradation of free RNA in our study. The search on PubMed revealed few results on the use of nuclease-like benzonase in viability assays of microorganisms. Two studies successfully applied enzymes to degrade free nucleic acids [33, 51]. Monteiro et al. performed a pretreatment of wastewater samples with the universal nuclease to measure the amount of infectious SARS-CoV-2 [51]. Amar et al. pretreated human skin microbiome samples with benzonase to remove NA of dead bacteria to ensure the sequencing of NA from live microbiota [33]. In another study to distinguish infectious from non-infectious human rotaviruses and noroviruses from fecal sample, benzonase application was not successful [31]. The author has no explanation for such effect.

The samples collected behind the air purifier of UV-C were completely inactivated. UV irradiation can damage the NA, while the protein coat remains intact and protects the NA [52]. This would prevent the enzymes from degrading the NA of these inactivated viruses. However, an UV exposure time exceeding 45 min increases the permeability of the envelope [53], in addition, high UV dose (≤1250 J/m^2^) are able to destroy the NA as well as the capsid [54].

In previous studies on the efficacy of air purifiers, a specific pathogen is often aerosolized and tested for infectivity at the end of the test procedure by culturing bacteria or viruses [18, 19, 39, 55, 56]. In our study, a rapid protocol to distinguish between intact infectious and distorted FCoV particles was developed to accelerate the efficacy testing, using a mixture of nucleases and real-time RT-PCR or RT-RAA. The nucleases removed free NA so that after NA extraction only NA from intact virus particles could be detected by molecular assays. The approach could also be carried out with other pathogens in the future, extending its use to other areas besides air purifier testing.
